# Identification of Heterotic Loci with Desirable Allelic Interaction to Increase Yield in Rice

**DOI:** 10.1186/s12284-021-00539-z

**Published:** 2021-11-26

**Authors:** Yin Xiong, Chaopu Zhang, Hongju Zhou, Wenqiang Sun, Peng Wang, Dianwen Wang, Xianjin Qiu, Jauhar Ali, Sibin Yu

**Affiliations:** 1 National Key Laboratory of Crop Genetic Improvement, College of Plant Science and Technology, Huazhong Agricultural University, Wuhan, 430070 China; 2 International Rice Research Institute, DAPO Box 7777, Metro Manila, Philippines

**Keywords:** Heterotic loci, Allelic interaction, Grain yield, Overdominance, *Ghd8*, Rice

## Abstract

Heterosis denotes the superiority of a hybrid plant over its parents. The use of heterosis has contributed significantly to yield improvement in crops. However, the genetic and molecular bases on heterosis are not fully understood. A large number of heterotic loci were identified for 12 yield-related traits in one parental population of chromosome segment substitution lines (CSSLs) and two test populations, which were interconnected by CSSLs derived from two rice genome-sequenced cultivars, Nipponbare and Zhenshan 97. Seventy-five heterotic loci were identified in both homozygous background of Zhenshan 97 and heterogeneous background of an elite hybrid cultivar Shanyou 63. Among the detected loci, at least 11 were colocalized in the same regions encompassing previously reported heterosis-associated genes. Furthermore, a heterotic locus *Ghd8*^NIP^ for yield advantage was verified using transgenic experiments. Various allelic interaction at *Ghd8* exhibited different heterosis levels in hetero-allelic combinations of five near-isogenic lines that contain a particular allele. The significant overdominance effects from some hetero-allelic combinations were found to improve yield heterosis in hybrid cultivars. Our findings support the role of allelic interaction at heterotic loci in the improvement of yield potential, which will be helpful for dissecting the genetic basis of heterosis and provide an optional strategy for the allele replacement in molecular breeding programs in hybrid rice.

**Supplementary Information:**

The online version contains supplementary material available at 10.1186/s12284-021-00539-z.

## Background

Heterosis or hybrid vigor refers to the phenomenon for which a hybrid markedly outperforms its parents. The use of heterosis in crops such as rice and maize have contributed significantly to the improvement of yield. Massive efforts have been made in exploring the genetic mechanisms of heterosis, leading to propose three main genetic models for heterosis (Chen 2013; Zhang et al. 2021), which include dominance (Xiao et al. 1995) and overdominance (Li et al. 2001; Gjuvsland et al. 2010; Larièpe et al. 2012) at a single-locus level and epistasis at two or more loci (Yu et al. 1997; Zhou et al. 2012). The dominance and overdominance effects reflected as an allelic interaction at a single locus in hybrids may result in an alteration of expression profiles or protein–protein interaction (Shao et al. 2019). At the same time, epistasis is referred to as the modification of a gene by one or several other genes (Birchler et al. 2010; Schnable and Springer 2013). However, the genetic and molecular mechanisms of heterosis in plants are not fully understood.

Numerous quantitative trait loci (QTLs) have been identified for heterosis in various plants (Li et al. 2001; Wang et al. 2012; Zhou et al. 2012; Huang et al. 2016; Zhu et al. 2016), indicating that complex genetic bases of heterosis. The advances of integrating multi-omics in the identification of heterotic loci or genes support the significant role of the allelic interactions at both single locus and multiple loci in plant heterosis (Huang et al. 2015; Li et al. 2016; Liu et al. 2020). For example, *SINGLE FLOWER TRUSS* (*SFT*) in tomato (*Lycopersicon esculentum* Mill.) is firstly identified as a single overdominant gene for yield (Krieger et al. 2010). In rice (*Oryza sativa* L.), *Heading date 3a* (*Hd3a*) has been identified to be responsible for heterosis in a large-scale F_2_ population (Huang et al. 2016). The gene *Ideal Plant Architecture1* (*IPA1*) that regulates plant architecture enhancing yield heterosis in rice, displays a strong overdominance effect from *IPA1* heterozygotes (Huang et al. 2016). Similarly, the ortholog gene of *IPA1* in maize (*Zea mays* L.), *unbranched 3* (*ub3*) has also been identified as the major candidate gene for heterosis advantage in three F_2_ populations (Liu et al. 2020). The other gene *OsMADS1* showed an incomplete dominance for grain size in rice and near-isogenic lines containing a particular alleles could highly increase grain yield by more than 8% (Wang et al. 2019a). Since the development of genome re-sequencing of germplasms allows us capture various allelic variations at any gene of interest, the intriguing issue arises of how to explore optional allelic combinations that could produce higher heterosis performance in hybrids. However, few cases where diverse effects of various allelic interactions at a heterotic locus have been reported.

Rice is the staple crop, contributing for nearly half of worldwide population’s food consumption (Elert 2014). The increase of rice yield is critical to ensuring global food security. Exploitation of interspecies hybrid vigor between *indica/xian* and *japonica/geng* has been a promising approach to enhance rice yield potential (Qian et al. 2016; Tao et al. 2016). The genetic dissection of hybrid vigor could facilitate this efficient exploitation. However, the precisely identification of heterotic loci has been limited in many segregating populations such as F_2_ and recombinant inbred lines because of some epistatic or high-order gene interactions in a complex genetic background (Yu et al. 1997; Li et al. 2001). With the advantage of chromosome segment substitution lines (CSSLs), each containing only one or a few introduced donor segments in the same background, the genetic effect and contribution of a gene can be confirmed without the effect of other loci in genome (Ali et al. 2010; Zhang et al. 2021). Thus, CSSLs and their derived populations could provide one of the optimal approaches for dissecting the genetic factor of heterosis at the single-locus level (Zhang et al. 2019). In this study, the objectives are to detect heterotic loci using CSSLs and their derived testing populations of backcross and testcross, and to unravel allelic interaction at a given heterotic loci using near-isogenic lines in rice. As a result, a number of heterotic loci (HLs) were identified for yield and yield-related traits in the backcross (BC) and testcross (TC) populations. Among them, a major heterotic gene (*Ghd8*) was verified through transgenic experiments. Moreover, different interaction effects arose from various allelic combinations of *Ghd8* were found associated with yield heterosis. Our findings of heterotic loci with favorable allelic combinations provide new insights into the genetic basis of heterosis. This will be useful for improving yield by hybrid rice breeding.

## Materials and Methods

### Plant Materials

Three mapping populations were developed and used in this study. The first population of 146 CSSLs was developed from a cross between two genome-sequenced rice cultivars, *japonica* Nipponbare (NIP) as the donor and elite *indica* Zhenshan 97 (ZS97) as the recurrent parent, using a backcross scheme of at least four times backcrossing with a marker-assisted selection (MAS) approach. The backcross population contained 146 F_1_ hybrids (CSSL × ZS97) derived by backcrossing each CSSL with the recurrent parent ZS97. The elite restorer line Minghui 63 (MH63) as the male parent was testcrossed with each CSSL to generate the testcross population. The parental lines (MH63, NIP, and ZS97) and hybrid cultivar Shanyou 63 (SY63, MH63 × ZS97) were used as controls in the phenotype experiments.

### Experimental Design and Phenotypic Evaluation

Two experiments were conducted to dissect of the genetic basis of heterosis in rice. First, three mapping populations were used to analyze the allelic effect at a single locus. A CSSL and BC were used to identify the mid-parent heterotic loci (HL_MP_) in the homozygous background of ZS97. At the same time, a TC along with a check (SY63) were used to detect the over-standard heterotic loci (HL_OS_) in a similar heterozygous background of SY63. Second, to determine the interaction of different allelic combinations at *Ghd8*, an half-diallel mating design was used to generate ten allelic combinations with five parental lines (NIL-*Ghd8*^ZS97^, NIL-*Ghd8*^NIP^, NIL-*Ghd8*^9311^, NIL-*Ghd8*^ACC10^, and NIL-*Ghd8*^MH63^). The five near-isogenic lines (NILs) carrying different *Ghd8* alleles were developed independently from the cross of four parents (NIP, 9311, ACC10, and MH63) as donors and ZS97 as the recurrent parent using a MAS backcross scheme. These NILs have the common background of ZS97.

All the lines were grown at the experimental station of Huazhong Agricultural University in Wuhan (30.48° N, 114.2° E), China. A randomized complete block design was carried out with two replications for three mapping populations in 2006 (E1) and 2007 (E2), respectively. The same field experimental design with three replications was used for the ten allelic combinations and NILs. Each line was planted in four rows with 10 individuals per row at a spacing of 16.7 × 26.6 cm. The eight plants in the middle of each row were harvested individually at maturity and used for scoring traits. The field was managed according to local standard practices.

Twelve quantitative traits were assayed: grain number (GN), heading date (HD), number of primary branches (PB), plant height (PH), panicle length (PL), panicles per plant (PP), panicle weight (PW), number of secondary branches (SB), spikelet number (SN), seed setting ratio (SS), thousand-grain weight (TGW), and grain yield per plant (YD).

### Transgenic Test of Ghd8 Effects on Heterosis

The transgenic experiments were performed to analyze *Ghd8* effects on heterosis. Homozygous complementary transgenic lines with the NIP alleles of *Ghd8* (*Ghd8*^NIP^) introduced into ZS97 (here named as NIL-*Ghd8*^ZS97^) were developed previously (Yan et al. 2011). An F_1_ hybrid was then generated by crossing each transgenic line with the corresponding negative control line or ZS97.

### DNA Extract and Genotype of Hybrids

Genomic DNA was extracted from young leaves using the CTAB method (Murray and Thompson, 1980) with minor modifications. Genotyping of the 146 CSSLs by using a RICE 6 K chip generated a total of 5,102 high-quality single nucleotide polymorphisms (SNPs), which were evenly distributed on all 12 chromosomes (Sun et al. 2015). A genetic bin map with 518 bins was constructed based on the recombination breakpoints in the CSSLs. The genotypes of the BC and TC were deduced from each corresponding CSSL. The insertion/deletion (Indel) marker PID2 (F: TAGAGATGAAATGGAGGTG; R: GTCTCATGTTCTTCAACATG) was used to identify the genotypes of all the allelic combinations, except that NIP/ACC10 hybrid was determined by Indel marker PID3 (F: CTTATCTATCAAGGTGCTC; R: TGCACACATGTAATGCAAAC), and MH63/ZS97 hybrid was identified by simple sequence repeat (SSR) marker RM5556 (F: GTAAGCCATTTGCACGGACAAGG; R: GAGCTCAGGATCATCCCTACATGC). PID2 was also used to identify the genotypes of complementary transgenic hybrids. Polymerase chain reaction was performed following the procedure of Panaud et al (1996). The Indel and SSR markers were separated by 4% polyacrylamide gel electrophoresis and visualized by silver staining.

### Data Analysis

The additive effect (*a*) was calculated using the following equation: *a* = (CSSL—ZS97)/2. The dominance effect (*d*) was estimated as *d* = F_1_—(CSSL + ZS97)/2. The mid-parent heterosis (MPH) was calculated as MPH = (F_1_—MP)/MP × 100%, MP = (CSSL + ZS97)/2, where F_1_ is the phenotypic value of the BC. The over-standard heterosis (OSH) value was calculated as OSH = (F_1_—SY63)/SY63 × 100%, where F_1_ is the phenotypic value of the TC. For allelic combinations at *Ghd8*, MPH = (F_1_—MP)/MP × 100%, MP = (NIL-*Ghd8*^*i*^ + NIL-*Ghd8*^*j*^)/2, where the F_1_ represented the phenotypic value of the hybrid for two NILs (NIL-*Ghd8*^*i*^ × NIL-*Ghd8*^*j*^, *i* and *j* denote different alleles of *Ghd8*). For complementary transgenic plants, the additive and dominance effects were calculated as *a* = (homozygous-positive transgenic line—negative line)/2, *d* = F_1_—(homozygous-positive transgenic line + negative line)/2. The estimated additive and dominance effects were used to calculate *|d/a|* for the classification of HL_MP_ as additive effect (A) (|d/a|< 0.2), partial dominance (PD) (0.2 ≤|d/a|< 0.8), complete dominance (CD) (0.8 ≤|d/a|< 1.2), and overdominance (OD) (|d/a|≥ 1.2), as described previously (Stuber et al. 1987). The means and standard error of the phenotypic values were analyzed in Microsoft Excel 2010.

### QTL Analyses

To decrease multicollinearity among the bin markers, the linear ridge regression method was used for the QTL analysis with the bin-map in the CSSLs as described previously (Sun et al. 2015) and for the QTL analysis of MPH and OSH values. A significance level of *P* < 0.05 was set as the threshold in the three mapping populations to declare the presence of a putative QTL in a given bin. If several adjacent bins showed *P* values lower than the threshold, the QTL was tentatively located in the bin (peak bin) with the lowest *P* value (Sun et al. 2015). The phenotypic variance explained by each QTL was decomposed using “relaimpo” package of R (“*lmg*” function). QTL nomenclature followed the principles suggested in a previous report by McCouch (2008).

## Results

### Phenotypic Performance of CSSLs, BC, and TC Populations

**Fig. 1:**
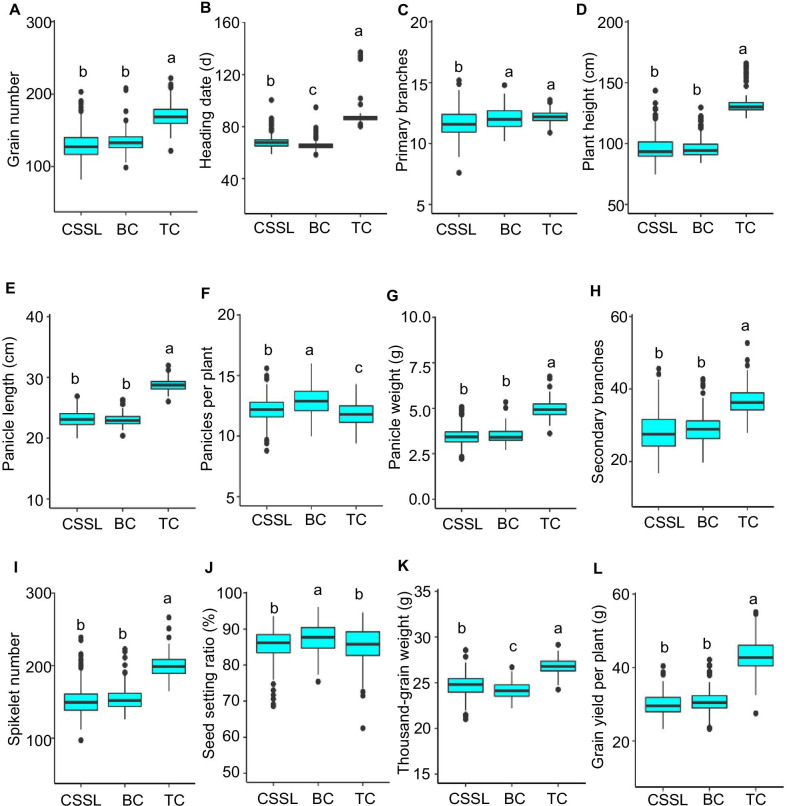
Boxplots of 12 yield-related traits among chromosome segment substitution lines (CSSLs), backcross population (BC), and testcross population (TC). Box edges indicate the range of the 25th to 75th percentiles, with the median value shown as the bold middle line. The whiskers represent the range of 5% to 95% of the data, and dots are outliers. The different lowercase letters in the boxplots denote significance among the medians of three populations at *P* < 0.05

**Fig. 2:**
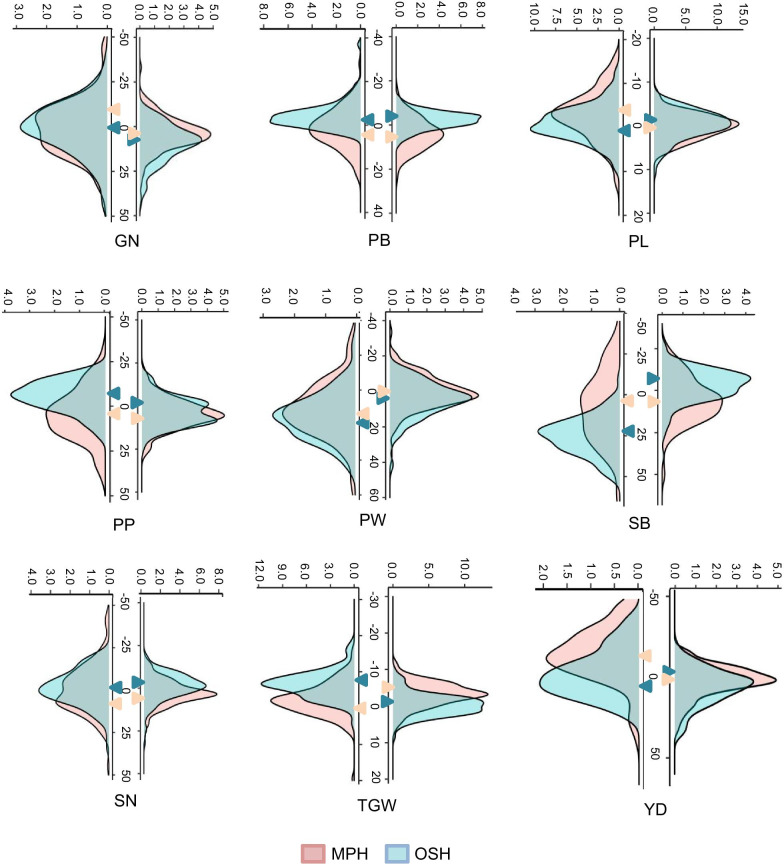
Distribution of mid-parent heterosis (MPH) and over-standard heterosis (OSH) for yield-related traits. The MPH and OSH of the yield-related traits were evaluated in 2006 (E1) and 2007 (E2), which are on the left and right of each plot, respectively. The y-axis represents the density (%). The x-axis represents the MPH value (%) and OSH value (%) for each trait. The median of each trait is indicated by the the colored triangle. GN, grain number; PB, number of primary branches; PL, panicle length; PP, panicles per plant; PW, panicle weight; SB, the number of secondary branches; SN, spikelet number; TGW, thousand-grain weight; YD, grain yield per plant

Three mapping populations (CSSLs, BC, and TC) exhibited wide phenotypic variances with continuous distribution for 12 yield-related traits (Fig. 1; Additional file 1: Table S1), showing a quantitative trait inheritance pattern. Most of the lines in the CSSLs and BC populations had similar phenotypic performance as ZS97, except for several lines that showed significantly higher or lower values than ZS97 (Fig. 1; Additional file 1: Table S1), indicating that these lines carry either the introduced homozygous NIP or heterozygous NIP segments associated with the measured traits. Moreover, wide variation and continuous distribution of MPH and OSH values were observed (Fig. 2; Additional file 1: Table S1). Several hybrids (of BC and TC) also exhibited heterosis values in two directions significantly higher or lower than corresponding controls for yield-related traits (Fig. 2). The TC population within the complex heterozygous background revealed similar variation in heterosis values to BC population within the background of ZS97 (Fig. 2; Additional file 1: Table S1).

Correlation analysis was performed for these 12 traits among three populations (Additional file 2: Figure S1). Significantly positive correlations were observed in all of the pairwise traits except for seed setting ratio between the CSSLs and BC. Similarly, significantly positive correlations were found in seven traits (HD, PB, PH, PL, SB, SN, and TGW) between the CSSL and TC. However, only four traits (HD, PH, PL, and SB) exhibited significantly positive correlations but with low values between the BC and TC. These results indicate that different genetic bases exist for trait performances among the three populations.

### Detection of QTLs in CSSLs

**Fig. 3:**
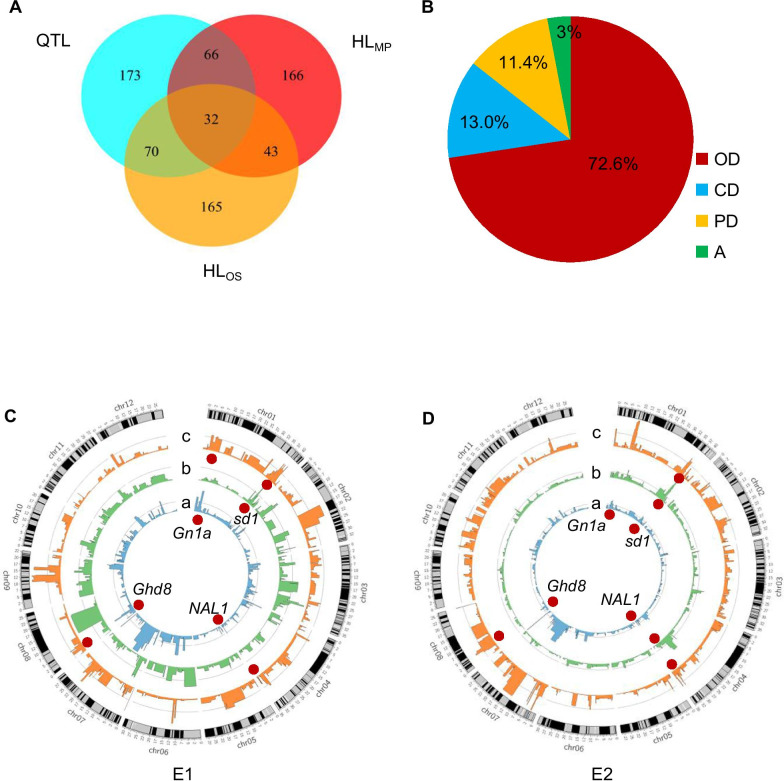
QTLs and heterotic loci for mid-parent heterosis (MPH) and over-standard heterosis (OSH) detected in the CSSLs, BC, and TC populations. **a** The quantitative trait loci (QTLs) and HLs were co-localized in the same or overlapping regions among the three populations. **b** The proportions of the different genetic effects at mid-parent heterotic loci (HL_MP_). OD, overdominance; CD, complete dominance; PD, partial dominance; A, additive. **c**, **d** Genome-wide distribution of QTLs, HL_MP_ and over-standard heterotic loci (HL_OS_) for spikelet number (SN) in 2006 (E1) and 2007 (E2). Rice chromosomes with bins are indicated in the outer circle. The circles from inward to outward show QTLs (**a**), HL_OS_ (**b**), and HL_MP_ (**c**), respectively. For each bar diagram, the *x*-axis represents the physical location along each numbered chromosome. The *y*-axis represents the *P* value for single nucleotide polymorphism (SNP) association. Dashed lines indicate the declaration thresholds. Some major heterosis-related genes reported in previous studies are indicated by red dots in **c**, **d**

The linear ridge regression method was used for the QTL mapping in CSSLs to decrease multicollinearity among markers. A total of 341 QTLs for 12 yield-related traits were identified in the CSSLs across two environments (E1 and E2) (Fig. 3a), and they were distributed on all 12 chromosomes (Additional file 1: Table S2). Among them, 114 QTLs were detected in both two environments and 40.4% of the loci suggested that the homozygous NIP alleles increased the phenotypic values. The total phenotypic variances ranging from 47.5 to 79.2% were explained by 14 to 24 QTLs for different traits (Additional file 1: Table S2). In the case of HD, 20 QTLs were detected across two-year trials; among them, *qHD7.4* on chromosome 7 had the largest effect, explaining 9.5% and 16.1% of the phenotypic variance in E1 and E2, respectively. Twenty-seven QTLs for PH were identified; of these, *qPH1.4* on chromosome 1 explaining 40.6% of the phenotypic variance, exhibited the greatest effect on PH. For panicle traits, 28, 24, 32, and 30 QTLs were identified for PB, PL, PP, and PW, respectively. Among them, the QTL regions (*qPB7.5*/*qPL7.6*/*qPW7.4*) on chromosome 7 (29.62–29.70 Mb) overlapped for three panicle traits with NIP alleles increasing the phenotypic values. For spikelet traits, 35, 25, and 27 QTLs were identified for GN, SB, and SN, respectively, of which three QTLs (*qGN7.6*/*qSB7.5*/*qSN7.4*) mapped on the same region (29.62–29.70 Mb) exhibited the most significant effect on these three traits in both two environments (Additional file 1: Table S2).

### Detection of HL_MP_ for Yield Traits

The CSSL and BC populations were used to identify HL_MP_ in the homozygous background of ZS97. Every line in BC population contains only one or a few introduced heterozygous NIP segments (NIP/ZS97) within the homozygous background of ZS97. Therefore, the HL_MP_ mainly present a non-additive genetic effect between NIP and ZS97 alleles at a given single locus. A total of 307 HL_MP_ were detected for 12 traits across two environments (Fig. 3a). Among them, 42 QTLs were detected in both two environments. Most of HL_MP_ (57.0%) increased MPH for yield and yield-related traits (Additional file 1: Table S3). For HD, 25 HL_MP_ were detected; among these, *qBHD2.2* and *qBHD7.4* had the most significant effect, explaining 10.3% and 12.9% of the phenotypic variance in E1 and E2, respectively. For PH, 32 HL_MP_ were identified; of these, *qBPH1.3* revealed the most significant effect, explaining 8.3% of the phenotypic variance. For panicle traits, 21, 31, 19, and 20 HL_MP_ were detected for PB, PL, PP, and PW across the two-year trials. Among them, the QTL overlapping region (*qBPL1.1/qBPW1.1/qBPB1.1*) on chromosome 1 (3.04–5.72 Mb) was detected with a positive effect on multiple panicle traits. Seven-three loci were identified for three spikelet traits across two environments, with 21 HL_MP_ for GN, 25 for SB, and 27 for SN. Among these, *qBSB8.1/qBSN8.1* on chromosome 8 (3.80–4.37 Mb) was detected in both two environments and was located in the same region of *Ghd8* that was reported to regulate heading date and grain number (Yan et al. 2011). For SS, 36 HL_MP_ were detected; among these, *qBSS9.2* had the most significant effect, explaining 6.8% of the phenotypic variance. A total of 28 HL_MP_ were detected for thousand-grain weight (TGW) in two environments. Among these, *qBTGW8.1* had the largest effect and explained 16.4% of the phenotypic variance in E1. Twenty-two HL_MP_ were detected for YD in E1 and E2 and 14 of them showed positive effects (Additional file 1: Table S3).

In addition, all three genetic components (additive, dominance, and overdominance effects) at HL_MP_ were estimated (Fig. 3b). The majority (97%) of HL_MP_ exhibited an overdominance or dominance effect (Fig. 3b; Additional file 1: Table S3). These data indicate that overdominance and dominance effects play a crucial role in MPH.

### Detection of HL_OS_ for Yield Traits

Each line in the TC population contains one or a few introduced heterozygous (NIP/MH63) segments in the otherwise uniform heterozygous background of a widely used hybrid cultivar SY63. Therefore, each HL_OS_ effect represents an interaction effect between NIP and MH63 alleles at a given locus. HL_OS_ detected in TC are summarized in Table S4 (Additional file 1: Table S4). A total 310 HL_OS_ were identified for the 12 traits across two environments, which were distributed on all 12 chromosomes. Fifty-seven loci were detected in both two environments. Among them, 55.4% of the loci showed that NIP/MH63 hetero-allelic interaction increased over-standard heterosis. Twenty-eight HL_OS_ for HD were detected across two environments. Twenty-five HL_OS_ affecting PH was identified; among these, *qTPH1.6* on chromosome 1 (38.10–38.47 Mb) had the largest effect, explaining 40.8% and 39.9% of the phenotypic variance in both E1 and E2, respectively. For panicle traits, 26, 27, 26, and 26 HL_OS_ were detected for PB, PL, PP, and PW, respectively. Among them, three HL_OS_ (*qTPL4.1*/*qTPW4.1*/*qTPB4.1*) were localized in the same region (19.60–19.89 Mb) of chromosome 4, with the heterozygote increasing the phenotypic values. For spikelet traits, a total of 22, 26, and 28 HL_OS_ were identified for GN, SB, and SN, respectively. Three loci (*qTGN1.4/qTSB1.2/qTSN1.4*) overlapped in the same region (38.10–38.47 Mb) and exhibited the largest effect, which explained 11.1%, 8.0% and 11.3% of the phenotypic variance of GN, SB, and SN in E2, with the heterozygote increasing the phenotypic values. Twenty-five loci were detected for SS, with 16 of them showing that the heterozygous alleles decreased the phenotypic values. For TGW, a major locus *qTTGW5.3* explained 8.5% of the phenotypic variance in E1. For YD, 24 HL_OS_ were identified, explaining 45.1% and 38.9% of the phenotypic variance in E1 and E2, respectively. *qTYD1.2* had the most significant effect (Additional file 1: Table S4), explaining 16.2% of the phenotypic variance.

### Positive Effect of Ghd8 on Yield Heterosis

**Fig. 4:**
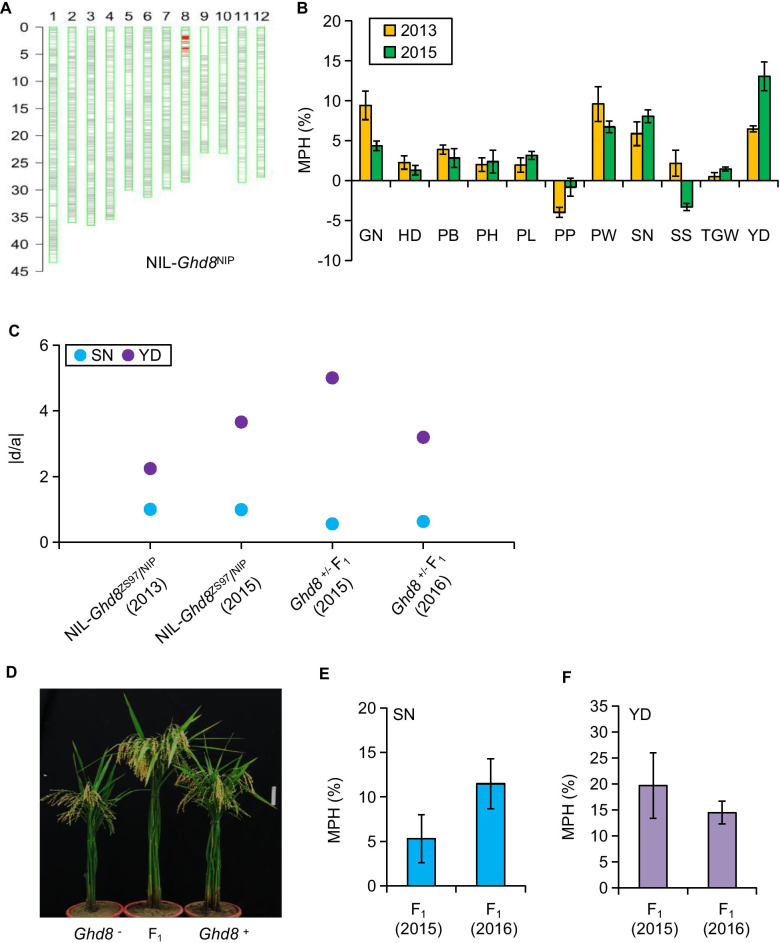
Validation of the *Ghd8* effect on mid-parent heterosis (MPH) using near-isogenic lines (NILs) and complementation transgenic lines. **a** Graphical genotype of NIL-*Ghd8*^NIP^ showing a single introduced Nipponbare segment encompassing *Ghd8* in the ZS97 background. **b** The heterosis advantage of *Ghd8* for the yield traits in hybrids of NIL-*Ghd8*^NIP^ and NIL-*Ghd8*^ZS97^ in a two-year trial. Error bar represents the mean ± SE (n = 3). **c** The dominance degree of *Ghd8* effect on spikelet number (SN) and grain yield per plant (YD) in hybrids. If the effect has an excessive degree of dominance, it is set to a score of 5.0. **d** Phenotypes of the complementation transgenic lines and their hybrids at maturity. *Ghd8*^+^, homozygous-positive transgenic line containing the alleles *Ghd8*^NIP^; *Ghd8*^−^, homozygous-negative control lines; F_1_ denotes the hybrid derived from the cross of the independent complementation transgenic plant and corresponding negative plant. Scale bars, 10 cm. **e**, **f** MPH of *Ghd8* for SN (E) and YD (F) in the F_1_ hybrids across a two-year trial. The error bar represents the mean ± SE (n = 3)

Among the HLs, a major loci on chromosome 8 (3.80–4.37 Mb) was identified for MPH and OSH of four traits (Fig. 3c, d; Additional file 1: Table S3-4), and it was located in the same region of the QTL for six yield-related traits detected in the CSSLs (Additional file 1: Table S2), which contains a known functional gene (*Ghd8*^NIP^) (Yan et al. 2011). To validate the effect of this HL, NIL-*Ghd8*^NIP^ that carries an introduced NIP segment encompassing *Ghd8* (Fig. 4a) was selected and crossed with NIL-*Ghd8*^ZS97^ to produce F_1_ hybrids. The MPH effects at *Ghd8* were assayed in 11 yield-related traits. The heterozygotes at *Ghd8* showed significant MPH for GN, PW, SN, and YD over the two parental lines (NIL-*Ghd8*^NIP^ and NIL-*Ghd8*^ZS97^) across two-year trials (Fig. 4b). As GN and PW are highly dependent on SS, but SS is easily affected by high temperature in summer during the experiments, a representative yield component, SN, which is highly correlated with GN and PW, is used as the example to assess the heterotic effect. The heterozygous *Ghd8* showed high MPH for YD (9.7%) and SN (6.9%) across the two-year trials (Fig. 4b). Moreover, an overdominance effect (*|d/a|*= 2.94) of *Ghd8* on YD and a complete dominance effect (|*d*/*a*|= 0.99) on SN were found in the NIL- *Ghd8*^ZS97^/NIL-*Ghd8*^NIP^ hybrid (Fig. 4c). These results confirm that *Ghd8* is a heterotic locus with an overdominance or dominance effect increasing yield and yield traits.

### Validation the Heterotic Effect of Ghd8 by Transgenic Experiment

To validate the heterotic effect of *Ghd8*, the complementary transgenic line (*Ghd8*^+^) carried the *Ghd8*^NIP^ alleles were crossed with NIL-*Ghd8*^ZS97^ (*Ghd8*^*−*^) to generate the heterozygote (named F_1_) containing a hetero-allelic combination (*Ghd8*^ZS97^ and *Ghd8*^NIP^ alleles) (Fig. 4d). The F_1_ hybrid increased average MPH of YD and SN by 17.1% and 8.4% across the two-year trials, respectively (Fig. 4e, f; Additional file 1: Table S5). For YD, the heterozygous genotype (*Ghd8*^*NIP*^/*Ghd8*^*ZS97*^) exhibited high MPH (e.g. 14.5% ~ 19.7%). The combination showed moderate heterosis for SN (e.g. 5.3% ~ 11.5%) (Fig. 4e, f; Additional file 1: Table S5). Moreover, the heterozygous *Ghd8* showed a positive overdominance effect on YD and partial dominance effect on SN (Fig. 4c; Additional file 1: Table S5). These results indicate that *Ghd8* affecting heterosis in yield components, and the two alleles (*Ghd8*^NIP^ and *Ghd8*^ZS97^) exhibited a strong interaction on MPH in the ZS97 background.

### Allelic Interaction of Ghd8 Associated with Heterosis

**Fig. 5:**
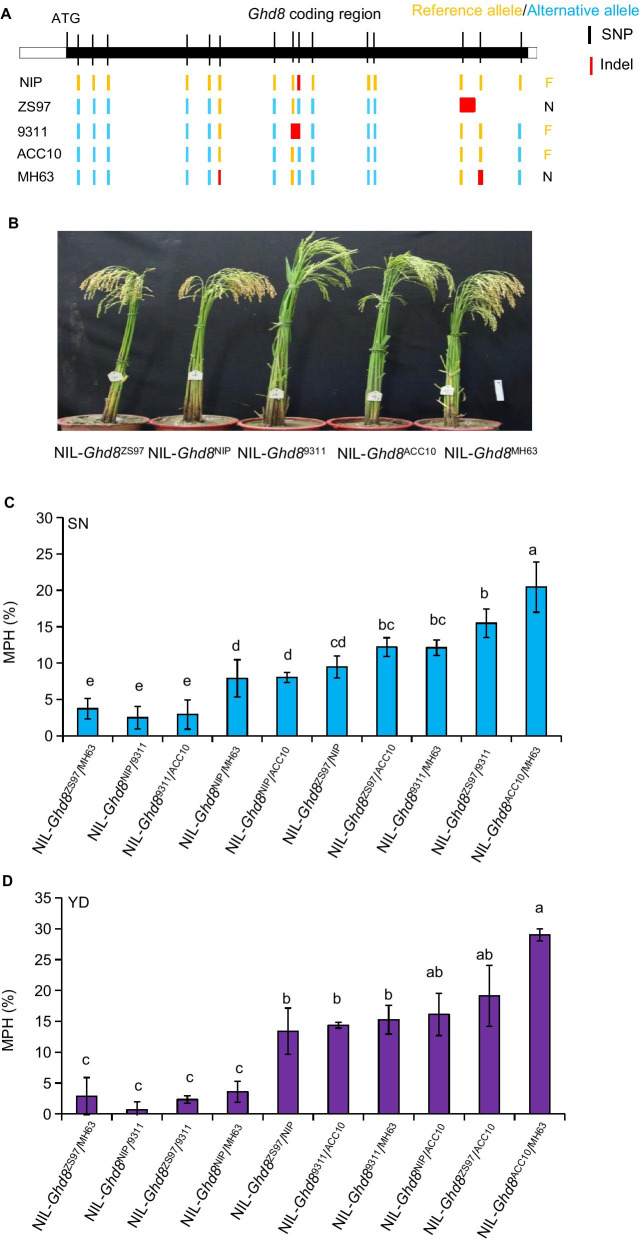
Yield mid-parent heterosis (MPH) of 10 allelic combinations at *Ghd8* in heterozygotes within the background of ZS97. **a** Schematic gene mode showing nucleotide variations in the coding region of *Ghd8* among five parents (NIP, ZS97, 9311, ACC10 and MH63). NIP (Nipponbare) is used as a reference. Polymorphic nucleotides are indicated by different color lines. “F” and “N” denote functional and non-functional alleles, respectively. **b** Plant image of five NILs (NIL-*Ghd8*^ZS97^, NIL-*Ghd8*^NIP^, NIL-*Ghd8*^9311^, NIL-*Ghd8*^ACC10^, and NIL-*Ghd8*^MH63^). The images were taken at the maturity of NIL-*Ghd8*^ZS97^; scale bar, 10 cm. **c**, **d** MPH of *Ghd8* for spikelet number (SN) and yield per plant (YD) in ten hybrids with different allelic combinations. The error bar represents the mean ± SE (n = 3). NIL-*Ghd8*^NIP^, NIL-*Ghd8*^9311^, NIL-*Ghd8*^ACC10^, and NIL-*Ghd8*^MH63^ represent those NILs carrying corresponding *Ghd8* alleles from different donors within the same ZS97 background, respectively. The different letters denote significant differences by LSD test at *P* < 0.05. The error bar represents the mean ± SE (n = 3)

In a previous study, three alleles (*Ghd8*^NIP^, *Ghd8*^9311^ and *Ghd8*^ACC10^) were reported to be functional, while *Ghd8*^ZS97^ and *Ghd8*^MH63^ being loss-of-function alleles due to a premature stop codon occurred (Fig. 5a) (Wang et al. 2019b). To investigate the interaction effect of *Ghd8* on yield MPH, five NILs (NIL-*Ghd8*^ZS97^, NIL-*Ghd8*^NIP^, NIL-*Ghd8*^9311^, NIL-*Ghd8*^ACC10^, and NIL-*Ghd8*^MH63^) were developed, with each carrying an introduced segment covering *Ghd8* from a particular donor in the same background of ZS97 (Fig. 5b), and a half-diallel mating design with five NILs that contain a particular allele (*Ghd8*^ZS97^, *Ghd8*^NIP^, *Ghd8*^9311^, *Ghd8*^ACC10^, and *Ghd8*^MH63^) was used to generate 10 hetero-allelic combinations. The combination (*Ghd8*^ACC10^/*Ghd8*^MH63^) revealed the highest MPH for YD (29.0%) and SN (20.4%) (Fig. 5c, d; Additional file 1: Table S6). Another five combinations (*Ghd8*^ZS97^/*Ghd8*^ACC10^, *Ghd8*^ZS97^/*Ghd8*^NIP^ *Ghd8*^9311^/*Ghd8*^ACC10^, *Ghd8*^9311^/*Ghd8*^MH63^, *Ghd8*^NIP^/*Ghd8*^ACC10^) showed significantly higher YD heterosis over the check *Ghd8*^ZS97^/*Ghd8*^MH63^ (Fig. 5d). However, three allelic combinations (*Ghd8*^NIP^/*Ghd8*^9311^, and *Ghd8*^ZS97^/*Ghd8*^9311^, *Ghd8*^NIP^/*Ghd8*^MH63^) produced low or no YD and/or SN advantage over the check *Ghd8*^ZS97^/*Ghd8*^MH63^ (Fig. 5c, d; Additional file 1: Table S6). These results indicate that the interaction of various *Ghd8* alleles could cause different heterosis levels of yield.

## Discussion

In the present study, we identified 98 QTLs for yield traits and HL_MP_ for MPH in the same or overlapping regions in both CSSLs and BC population with the homozygous background of ZS97 (Fig. 3a). These commonly detected loci are in the accordance with the high correlations among the yield-related traits (Additional file 2: Fig. S1). As comparison, a larger number of loci were found only in the CSSLs not in the BC or TC (Fig. 3a; Additional file 1: Table S2-S4), suggesting that most HLs were caused by allelic interaction from heterozygotes. These results also suggest that independent genetic effects (*a*, *d*) at that loci are reflected in the homozygous NIP in CSSLs and heterozygous NIP alleles in BC. Seventy-five heterotic loci (HL_OS_) were found co-localized in the same or overlapping regions of HL_MP_, indicating that both heterozygous NIP/ZS97 and NIP/MH63 alleles at the detected loci significantly affect heterosis in rice, although the interaction effects were influenced by different genetic backgrounds. Of these heterotic loci for MPH and OSH, 32 also revealed major effects on the trait performances in the CSSLs (Fig. 3a).

Under comparison of the QTLs detected in the CSSLs with those in previous reports, at least 81 loci co-localized in the same or overlapping regions harboring the genes associated with yield-related traits (Additional file 1: Table S2). For example, *qPH1.4* for PH detected in the CSSLs was located near *sd1*, a gibberellin synthesis gene (*OsGA20ox2*) regulating plant height (Sasaki et al. 2002). Five loci (*qGN7.2/qHD7.1/qPH7.2/qSN7.2/qYD7.1*) were localized in the region that contains the known yield gene *Ghd7* (Xue et al. 2008). Five QTLs (*qGN7.6/qHD7.4/qPH7.5/qSN7.6/qYD7.5*) were mapped in the same region where *Ghd7.1* was reported to affect grain number, plant height, and heading date (Yan et al. 2013). Six loci (*qGN8/qPB8/qPH8.1/qPW8.1/qSB8/qSN8.1*) were detected in the region encompassing *Ghd8*, a yield-related gene with a pleiotropic effect on grain number, plant height, and heading date (Yan et al. 2011).

We have further identified that 42 HL_MP_ and 59 HL_OS_ located in the same or overlapping regions, where harbor many heterotic genes previously reported associated with yield-related traits (Additional file 1: Table S3-S4), such as the genes *Gn1a*, *LAX1*, *sd1*, *OsMADS22*, *NAL1*, *Hd1*, *Ghd7*, *Ghd7.1*, *Ghd8*, *IPA1*, and *Ehd1* (Huang et al. 2015, 2016; Li et al. 2016). Particularly, three HL_OS_ (*qTHD7.1/qTPW7.2/qTYD7.2*) were co-localized in the region of *Ghd7*, which was reported exhibiting a strong heterotic effect on heading date and spikelet number in hybrids (Liu et al. 2015; Huang et al. 2016). Three HL_MP_ (*qBPH7.4/qBHD7.4/qBSN7.4*) and four HL_OS_ (*qTHD7.4/qTPH7.2/qTSB7.3/qTYD7.4*) were all co-localized in the *Ghd7.1* region. Three HL_OS_ (*qTPB1.1/qTSN1.2/qTPW1.1*) were identified overlapping in the same region of *Gn1a*, which was reported as a grain number gene (Ashikari et al. 2005). Three HL_OS_ (*qTPL1.3/qTPW1.3/qTSN1.3*) were mapped in the region surrounding a lax panicle gene, *LAX1* (Komatsu et al. 2011). Three HL_MP_ (*qBPW10/qBPH10.2/qBHD10.2*) were mapped in the region containing *Ehd1*, which is a flowering time gene (Doi et al. 2004). Thus, the data on heterotic loci encompassing candidate genes associated with yield or yield components in both homozygous and heterozygous backgrounds could be immediately exploited for improving yield heterosis in hybrid rice breeding programs.

Notably, many studies reported that *Ghd8* could be an important candidate gene that affects heterosis for yield-related traits (Li et al. 2016; Huang et al. 2015, 2016; Chen et al. 2019; Lin et al. 2020). However, it lacked transgenic validation. In the present study, we identified four HL_MP_ (*qBHD8.1/qBPL8.2/qBSB8.1/qBSN8.1*) and one HL_OS_ (*qTHD8.1*) (Additional file 1: Table S3-S4) that were commonly detected in the *Ghd8* region and validated them as the major heterotic locus for yield and spikelet number using transgenic experiments (Fig. 4). Moreover, the interaction effects arose from some hetero-allelic combinations of *Ghd8* caused different levels of YD and SN heterosis. Three functional alleles (*Ghd8*^*NIP*^, *Ghd8*^*9311*^, and *Ghd8*^*ACC10*^), when interacting with non-functional allele *Ghd8*^*ZS97*^, significantly increased heterosis for YD and SN compared with the combination of two non-functional alleles (*Ghd8*^*ZS97*^ and *Ghd8*^*MH63*^) (Fig. 5). The hetero-allelic combinations, such as *Ghd8*^*ACC10*^/*Ghd8*^*MH63*^ and *Ghd8*^*9311*^/*Ghd8*^*MH63*^, also exhibited much significantly higher MPH for YD and SN than the check combination *Ghd8*^ZS97^/*Ghd8*^*MH63*^. These data suggest that the allelic interaction effect arose from the functional and non-functional alleles at *Ghd8* could produce more spikelet number and grain yield in heterozygotes than in the corresponding homozygotes or the check combination. However, the molecular mechanisms for the varied heterosis levels led by allelic combinations require further investigation. It has been reported that various allelic interactions may lead to novel hybrid expression patterns (He et al. 2010; Groszmann et al. 2015; Shao et al. 2019), protein metabolism (Goff, 2011; Chen, 2013), and epigenetic changes such as small RNAs and histone modification (Springer and Stupar, 2007; Lauss et al. 2019). In this regard, the transcriptional or post-transcriptional regulations, or polymer/dimer products from the allelic interaction may be causes of the heterosis variation. The developed NILs each contains a particular allele at the heterotic locus will provide an excellent stock to dissect the underlying mechanisms.

In addition, the elite rice hybrid SY63 has been successfully used for commercial hybrid production with the largest cultivated area in China during the past three decades (Xie and Zhang, 2018). In the present study, we found that the original hetero-allelic combination *Ghd8*^ZS97^/*Ghd8*^MH63^ in SY63 did not contribute to yield heterosis. However, the hetero-allelic combinations between *Ghd8*^ZS97^ (or *Ghd8*^MH63^) and any other alleles like *Ghd8*^NIP^, *Ghd8*^9311^, or *Ghd8*^ACC10^ in heterozygotes could produce a significant positive yield heterosis compared with the combination between *Ghd8*^ZS97^ and *Ghd8*^MH63^. Thus, the replacement of either allele *Ghd8*^ZS97^ or *Ghd8*^MH63^ of SY63 with those identified desirable *Ghd8* alleles with a marker-assisted selection approach can be used to improve the yield potential of hybrid cultivars.

## Conclusion

A large number of HLs for yield-related traits were identified using three rice CSSL interconnected populations. Of these loci, *Ghd8* was validated as a major HL for spikelet number and grain yield by transgenic experiments. Moreover, the investigation of 10 hetero-allelic combinations at *Ghd8* exhibited several desirable allelic interactions in heterozygotes that can enhance yield heterosis. These data provide new insights into understanding the genetic basis of heterosis and will be exploited for increasing yield potential in hybrid rice breeding programs to meet the demand of growing population.

## Supplementary Information

**Additional file 1 Table S1**. Phenotypic performances of 12 traits among the parents, CSSLs, BC, and TC across two environments. **Table S2**. QTLs were detected for 12 traits in CSSLs across two-year trials. **Table S3**. Mid-parent heterotic loci (HL_MP_) were detected for 12 traits in BC across two environments. **Table S4**. Over-standard heterotic loci (HL_OS_) were detected for 12 traits in TC population across two environments. **Table S5**. The degree of dominance and mid-parent heterosis for spikelet number and grain yield in transgenic hybrids. **Table S6 ** The degree of dominance and mid-parent heterosis for spikelet number and grain yield in 10 allelic combinations.

**Additional file 2 Figure S1**. Correlation coefficients for 12 traits among CSSLs, BC and TC populations in 2007. GN, grain number; HD, heading date; PB, number of primary branches; PH, plant height; PL, panicle length; PP, panicles per plant; PW, panicle weight; SB, the number of secondary branches; SN, spikelet number; SS, seed setting ratio; TGW, thousand-grain weight; and YD, grain yield per plant.

## Data Availability

The data sets supporting the results of this article are included within the article and its supporting files.

## Abbreviations

A: Additive effect
BC: Backcross
CD: Complete dominance
CSSLs: Chromosome segment substitution lines
HLs: Heterotic loci
HL_MP_: Mid-parent heterotic loci
HL_OS_: Over-standard heterotic loci
Indel: Insertion/deletion
MPH: Mid-parent heterosis
NIL: Near-isogenic line
OD: Overdominance
OSH: Over-standard heterosis
PD: Partial dominance
QTLs: Quantitative trait loci
SNP: Single nucleotide polymorphism
SSR: Simple sequence repeat
TC: Testcross
