# Supplementary material for: Identification of Heterotic Loci with Desirable Allelic Interaction to Increase Yield in Rice
Source: Rice (N Y). 2021 Nov 26;14:97. doi: 10.1186/s12284-021-00539-z (PMC8626550; doi:10.1186/s12284-021-00539-z)
Supplement: Supplementary file 2 — Additional file 2 Figure S1. Correlation coefficients for 12 traits among CSSLs, BC and TC populations in 2007. GN, grain number; HD, heading date; PB, number of primary branches; PH, plant height; PL, panicle length; PP, panicles per plant; PW, panicle weight; SB, the number of secondary branches; SN, spikelet number; SS, seed setting ratio; TGW, thousand-grain weight; and YD, grain yield per plant. [file 12284_2021_539_MOESM2_ESM.pdf]

Identification of heterotic loci with desirable allelic interaction to increase yield in rice

Yin Xiong[1†], Chaopu Zhang[1†], Hongju Zhou[1†], Wenqiang Sun[1], Peng Wang[1], Dianwen Wang[1], Xianjin Qiu[1], Jauhar Ali[2], Sibin Yu[1]*

[a] National Key Laboratory of Crop Genetic Improvement, College of Plant Science and Technology, Huazhong Agricultural University, Wuhan 430070, China

[b] International Rice Research Institute, DAPO Box 7777, Metro Manila, Philippines

* Correspondence author*:* Sibin Yu (ysb@mail.hzau.edu.cn)

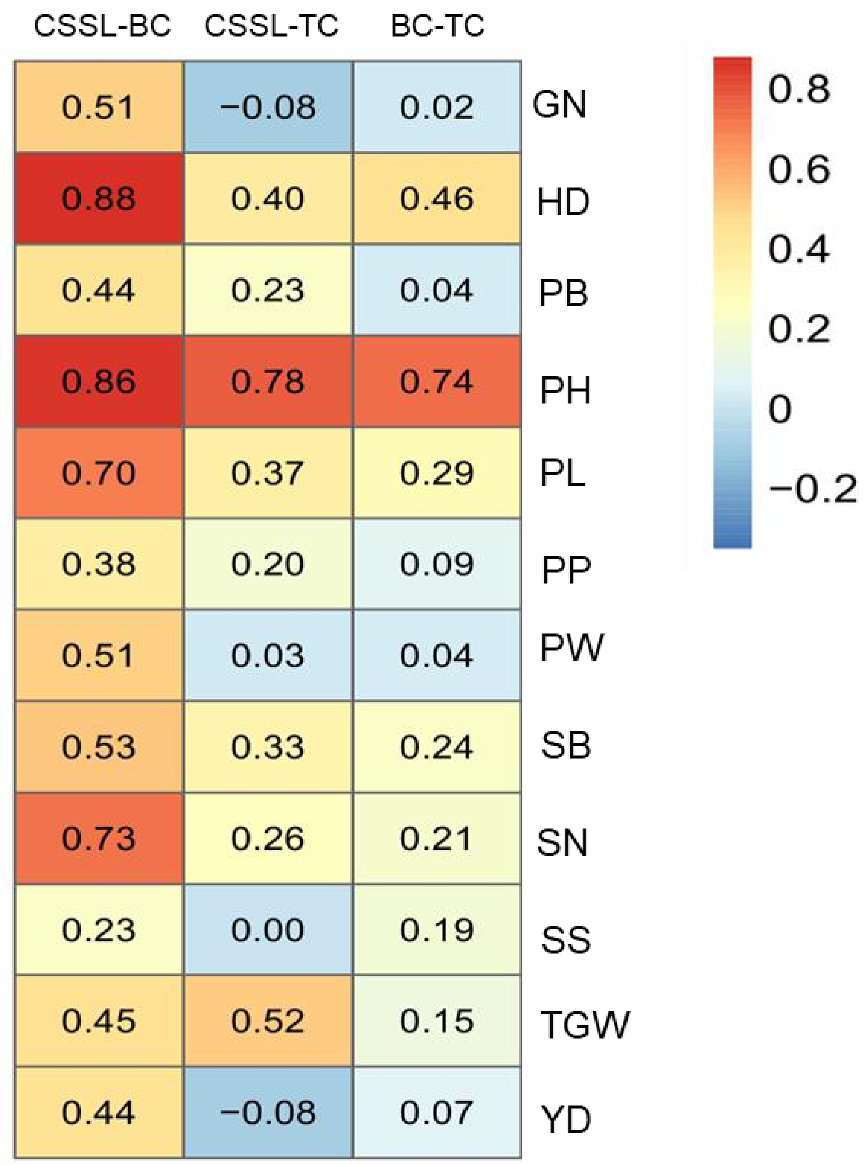

Fig. S1. Correlation coefficients for 12 traits among CSSLs, BC and TC in 2007. GN, grain number; HD, heading date; PB, number of primary branches; PH, plant height; PL, panicle length; PP, panicles per plant; PW, panicle weight; SB, the number of secondary branches; SN, spikelet number; SS, seed setting ratio; TGW, thousand-grain weight; and YD, grain yield per plant.
